# Can inhaled fluticasone alone or in combination with salmeterol reduce systemic inflammation in chronic obstructive pulmonary disease? – study protocol for a randomized controlled trial [NCT00120978]

**DOI:** 10.1186/1471-2466-6-3

**Published:** 2006-02-06

**Authors:** Don D Sin, SF Paul Man, Darcy D Marciniuk, Gordon Ford, Mark FitzGerald, Eric Wong, Ernest York, Rajesh R Mainra, Warren Ramesh, Lyle S Melenka, Eric Wilde, Robert L Cowie, Dave Williams, Roxanne Rousseau

**Affiliations:** 1 Department of Medicine (Respiratory Division), University of British Columbia, Vancouver, Canada; 2 Department of Medicine, University of Saskatchewan, Saskatoon, Canada; 3 Department of Medicine, University of Alberta, Edmonton, Canada; 4 Wetaskiwin General Hospital, Wetaskiwin, Canada; 5 Department of Medicine, University of Calgary, Calgary, Canada; 6 Lions Gate Hospital, North Vancouver, Canada; 7 Royal Alexandra Hospital, Edmonton, Canada; 8 Grey Nuns Hospital, Edmonton, Canada; 9 Lethbridge General Hospital, Lethbridge, Canada; 10 Matsqui-Sumas-Abbotsford General Hospital, Abbotsford, Canada

## Abstract

**Background:**

Systemic inflammation is associated with various complications in chronic obstructive pulmonary disease including weight loss, cachexia, osteoporosis, cancer and cardiovascular diseases. Inhaled corticosteroids attenuate airway inflammation, reduce exacerbations, and improve mortality in chronic obstructive pulmonary disease. Whether inhaled corticosteroids by themselves or in combination with a long-acting β_2_-adrenoceptor agonist repress systemic inflammation in chronic obstructive pulmonary disease is unknown. The Advair Biomarkers in COPD (ABC) study will determine whether the effects of inhaled corticosteroids alone or in combination with a long-acting β_2_-adrenoceptor agonist reduce systemic inflammation and improve health status in patients with chronic obstructive pulmonary disease.

**Methods/Design:**

After a 4-week run-in phase during which patients with stable chronic obstructive pulmonary disease will receive inhaled fluticasone (500 micrograms twice daily), followed by a 4-week withdrawal phase during which all inhaled corticosteroids and long acting β_2_-adrenoceptor agonists will be discontinued, patients will be randomized to receive fluticasone (500 micrograms twice daily), fluticasone/salmeterol combination (500/50 micrograms twice daily), or placebo for four weeks. The study will recruit 250 patients across 11 centers in western Canada. Patients must be 40 years of age or older with at least 10 pack-year smoking history and have chronic obstructive pulmonary disease defined as forced expiratory volume in one second to vital capacity ratio of 0.70 or less and forced expiratory volume in one second that is 80% of predicted or less. Patients will be excluded if they have any known chronic systemic infections, inflammatory conditions, history of previous solid organ transplantation, myocardial infarction, or cerebrovascular accident within the past 3 months prior to study enrolment. The primary end-point is serum C-reactive protein level. Secondary end-points include circulating inflammatory cytokines such as interleukin-6 and interleukin-8 as well as health-related quality of life and lung function.

**Discussion:**

If inhaled corticosteroids by themselves or in combination with a long-acting β_2_-adrenoceptor agonist could repress systemic inflammation, they might greatly improve clinical prognosis by reducing various complications in chronic obstructive pulmonary disease.

## Background

Chronic obstructive pulmonary disease (COPD) represents an increasing burden worldwide and is reported to be the sixth leading cause of death in 1990 [1] and the fourth in 2000 [2]. Discouragingly, by the year 2020, its mortality rate will rank third, only behind stroke and heart disease [1]. Although these figures are alarming, they are likely to be gross underestimates of the true health and economic burdens of COPD because COPD is an important risk factor for other causes of morbidity and mortality, including cardiovascular disorders and cancer [3,4].

While the pathobiology of COPD has not been fully elucidated, there is a growing recognition that systemic inflammation may play a salient role in COPD progression and morbidity [5]. There is now a large body of data that show systemic inflammation exists in stable COPD and that the intensity of the inflammatory process relates to the severity of the underlying disease [6]. Systemic inflammation has been linked with the extra-pulmonary manifestations in COPD including sudden deaths, arrhythmias, strokes, myocardial infarction, and cancer independent of confounding factors such as age and smoking [4,6,7]. Additionally, in COPD, systemic inflammation has been associated with diminished peripheral muscle strength, reduced exercise endurance, workload, and six-minute walk distance, poor health status, and quality of life [8]. These data suggest that systemic inflammation may be intrinsically involved in the downward spiral in the health status of COPD patients. These data also raise the possibility that treatments modulating systemic inflammation could improve important health outcomes in COPD.

Inhaled corticosteroids are powerful but non-specific anti-inflammatory agents commonly used to treat patients with COPD [9]. Recent studies indicate that although inhaled corticosteroids do not alter the rate of decline in lung function, they reduce bronchial hyperreactivity, decrease the frequency of exacerbations, and slow the rate of decline in the patient's health status [10,11]. A pooled analysis of clinical trial data indicates they reduce mortality by ~25% relative to placebo over three to four years of follow-up [12]. Interestingly, most of the beneficial signal associated with inhaled corticosteroids was observed in a subgroup of COPD patients with moderate to severe disease [12]. In patients with mild disease, there was no survival advantage. These findings are consistent with data on exacerbations, which also demonstrate significant reductions related to inhaled corticosteroids only in those with moderate to severe disease [13]. Intriguingly, biomarkers of lung and systemic inflammation are increased in COPD patients with moderate to severe disease; patients with mild disease generally do not have increased levels of inflammatory biomarkers [14].

Systemic corticosteroids reduce serum C-reactive protein (CRP) and other circulating inflammatory cytokine levels in COPD [15]. They also down regulate certain inflammatory cells and cytokine levels in the airways of COPD patients [16]. Whether inhaled steroids can reduce circulating CRP levels in stable COPD is less certain. A pilot randomized clinical trial of 41 patients with COPD indicated that withdrawal of inhaled corticosteroids increased serum CRP levels by ~70%; whereas a two week treatment with inhaled fluticasone reduced CRP levels by ~50% [15]. These findings are consistent with a recently published observational study by Pinto-Plata and colleagues, who found that users of inhaled corticosteroids had serum CRP levels that were on average ~40% lower than those among corticosteroid non-users [17].

Recent experimental evidence indicates that long-acting β_2_-adrenoceptor agonists on their own can promote translocation of the glucocorticoid receptor complexes from the cytosol to the nucleus, a process that is critical in effecting the anti-inflammatory effects of corticosteroids [18]. When long-acting β_2_-adrenoceptor agonists are added to corticosteroids, the rate of glucocorticoid receptor translocation may be accelerated [19]. Moreover, Zhu and colleagues showed in a randomized controlled trial of 140 patients with moderate to severe COPD, that 12 weeks of treatment with salmeterol plus fluticasone decreased tissue expression of tumor necrosis factor (TNF)-α and interferon (IFN)-γ in the larger airways. They also found that combination therapy decreased sputum eosinophil (p = 0.03) and subepithelial mast cell counts (p = 0.08) in the bronchial tissues [20,21]. Importantly, these cellular changes were accompanied by a reduction in the rate of exacerbations in the combination therapy arm of the study. These results demonstrate that combination therapy decreases local inflammation in the lung, but whether combination therapy reduces systemic inflammation is still unknown.

### Aims of the study

The primary aim of this randomized, placebo-controlled, double-blind, multi-center clinical trial involving 11 centers in Western Canada is to determine whether inhaled corticosteroids alone or in combination with a long acting β_2_-adrenoceptor agonist reduce systemic inflammation in stable COPD.

### Specific objectives

This study will determine whether: 1) fluticasone alone or in combination with salmeterol lowers CRP levels by at least 30% below that achieved by placebo; and 2) combination therapy is better than fluticasone alone in reducing CRP levels. We will also determine whether: 1) CRP reductions are associated with improved health-related quality of life; 2) CRP reductions are associated with improved forced expiratory volume in one second (FEV_1_), and 3) inhaled fluticasone alone or in combination with salmeterol can also reduce interleukin (IL)-6 and other pro-inflammatory biomarkers in COPD.

## Methods/design

### Overview of the study design

**Figure 1:**
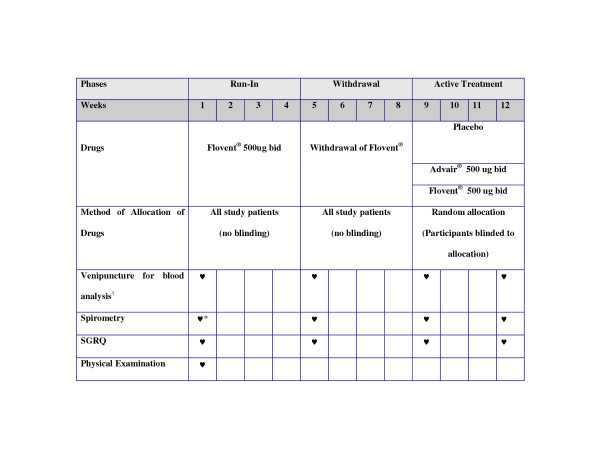
Summary of the Trial Protocol. * Post bronchodilator values; all else will be pre-bronchodilator values. † Must be done prior to initiation of therapy and completion of therapy or during periods of acute exacerbation or infection. For those exacerbations or infections requiring oral steroids and/or antibiotics, patients will be asked to return 2 weeks after completing their acute exacerbation or infection treatment.

This trial is a double blind study comparing the effects of inhaled fluticasone alone or in combination with salmeterol against placebo on serum CRP levels in COPD. All study participants will first undergo a run-in phase during which they will be treated with fluticasone 500 mcg bid for 4 weeks. This will be followed by a withdrawal phase wherein all participants will be free of any inhaled corticosteroids or theophylline products for 4 weeks. After the withdrawal phase, participants will be randomly assigned to one of three arms: placebo; inhaled fluticasone (500 mcg bid); or inhaled fluticasone/salmeterol combination (500/50 mcg bid). Randomization will be carried out centrally according to a computer-generated sequence stratified according to current smoking status with allocation concealment in a 1 (placebo arm) to 2 (fluticasone arm) to 2 (combination arm) distribution ratio. In this phase, all participants and study personnel will be blinded to the treatment assignment. This phase will last for 4 weeks. For safety reasons, during severe exacerbations (i.e. those requiring hospitalization or emergency visit for COPD), the treating physician can break the "code" and place study participants on medications needed to treat the exacerbation (See Figure 1 for protocol summary)

### Details of the run-in phase (4 weeks)

During this phase, all pulmonary medications with significant anti-inflammatory effects (e.g. inhaled corticosteroids, theophyllines, and leukotriene modifiers) will be discontinued. All participants will be maintained on inhaled fluticasone (500 mcg bid; Flovent Diskus^®^, GlaxoSmithKline Canada, Mississauga, ON). Short-acting β_2_-adrenoceptor agonists (e.g. salbutamol) and/or anticholinergic (ipratropium) will be allowed as rescue medications. Long-acting anticholinergic medication (i.e. tiotropium) will also be permitted, if clinically indicated as judged by the participants' attending physician. Long-acting β_2_-adrenoceptor agonists, however, will be prohibited.

### Details of the withdrawal phase (4 weeks)

During this phase, inhaled fluticasone will be discontinued and participants will not be allowed to take any other inhaled corticosteroids, theophyllines or leukotriene modifiers. Short-acting β_2_-adrenoceptor agonists (e.g. salbutamol) and/or anti-cholinergic (ipratropium) inhalers will be allowed as rescue medications. Long-acting anticholinergic inhaler (i.e. tiotropium) will be permitted as maintenance medication. Long-acting β_2_-adrenoceptor agonists will be prohibited.

### Details of the active treatment phase (4 weeks)

All participants will be randomized to one of 3 arms of the trial: placebo (placebo diskus, GlaxoSmithKline Canada, Mississauga, ON), inhaled fluticasone (500 mcg bid; Flovent Diskus^®^, GlaxoSmithKline Canada, Mississauga, ON) or inhaled fluticasone/salmeterol (500/50 mcg bid; Advair Diskus^® ^GlaxoSmithKline Canada, Mississauga, ON). Short-acting β_2_-adrenoceptor agonists (e.g. salbutamol) and/or anti-cholinergic inhaler (ipratropium) will be allowed as rescue medications. Long-acting anticholinergic medication (i.e. tiotropium) will be allowed as maintenance medication if clinically indicated as judged by the participants' attending physician. Any other long-acting β_2_-adrenoceptor agonists or inhaled corticosteroids will not be allowed. As well, theophyllines or leukotriene modifiers will not be permitted in this phase.

### Exacerbations during the study period (see figure 2)

**Figure 2:**
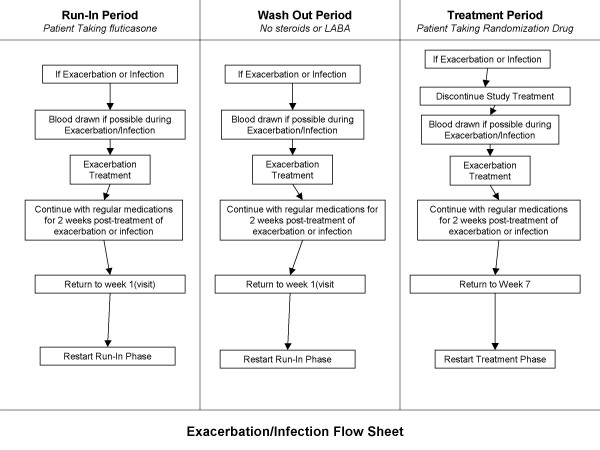
Management of Exacerbations during the Study. Abbreviations: LABA, long-acting β_2 _adrenoceptor agonist; SGRQ, St. George's Respiratory Questionnaire.

The participants will be instructed at enrollment to contact the local study coordinator at the earliest opportunity if their usual COPD symptoms (i.e. cough, dyspnea, sputum production, or sputum color change) worsen, or if they develop a fever (of unknown source), or active infections elsewhere (e.g. sinusitis). During exacerbations, study coordinators will see the participants as soon as possible and will codify all exacerbations according to Paggiaro's criteria [22]. A mild episode is one that can be self-managed by the patient at home, requiring only intensification of current therapy; a moderate event is defined as requiring antimicrobial and/or oral corticosteroid therapy; and severe is defined as an event requiring admission to a hospital or an emergency department. Treatment decisions during exacerbations will be rendered by the site (principal) investigators using the Canadian Thoracic Society guidelines in consultation with the participants' primary care provider [23]. Blood samples will be obtained at the time of the exacerbation. If participants do not or can not contact study coordinators during severe exacerbations, the study coordinator will make every attempt to obtain blood samples from the patients at the time of their presentation to the emergency department or hospital. For those who experience an exacerbation requiring systemic corticosteroids and/or antimicrobials, the study patients will be asked to return 2 weeks after they finish their course of exacerbation medications at which point their blood, spirometry, and health status measurements will be collected. Once clinical stability is achieved for a minimal of 2 weeks, the participants will resume study participation in accordance to the schedule published in figure 2.

### Study participants

All participants must have a clinical diagnosis of COPD as defined by the Global Initiative for Chronic Obstructive Lung Disease (GOLD) guidelines [24]. Spirometric criteria will include a FEV_1 _of less than 80% of predicted with an FEV_1 _to forced vital capacity (FVC) ratio of less than 0.70 (post-bronchodilator values). Additional inclusion criteria will be a cigarette smoking history of more than 10 pack-years, clinical stability as defined by the absence of exacerbations for at least 4 weeks, and age ≥ 40 years. Exclusion criteria will be any known disseminated malignancy, known chronic systemic infections or inflammatory conditions (e.g. rheumatoid arthritis, systemic lupus erythematosis, active sarcoidosis), previous solid organ transplantation, myocardial infarction or cerebrovascular accident within the past 3 months prior to study enrolment, females of child-bearing age (i.e. must be amenorrheic for at least 12 months), participation in a drug trial within the past 4 weeks prior to study enrolment, any subject who is unlikely to survive more than 6 months, recent upper respiratory tract infection within the 4 weeks prior to enrolment, unable to follow instructions, patients on chronic oral theophyllines and unable or unwilling to come off theophyllines for the study period, oral corticosteroids, or long-term immunosuppressive agents.

### Laboratory & other measurements

#### Blood collection

During every visit (and in the case of an exacerbation and/or infection according to Figure 2), study personnel will take two 10 ml collection of blood from participants through venipuncture using standard techniques. One sample (~10 mls) will be collected in tubes that do not contain an anti-coagulant and another in a tube that does contain an anti-coagulant. The tubes without the anti-coagulant will be allowed to clot for at least 30 minutes, then centrifuged at ≥ 1500 × *g *for 15 min at room temperature and then divided into aliquots (at least 2 serum samples) using a sterile plastic transfer pipet. The serum aliquots will be placed into supplied microtubes containing an anti-protease. Until used, the tubes containing the anti-protease will be stored in a -20°C freezer. The serum samples mixed with the anti-protease will be temporarily stored in a portable cooler containing frozen gel-packs and refrigerated at 4°C. Same-day shipment of the serum samples will be organized by the participating site to the central core laboratory located James Hogg iCAPTURE Centre for Cardiovascular and Pulmonary Research, St. Paul's Hospital, Vancouver, British Columbia, using a courier service, guaranteeing arrival within 24 hours of shipment. The tube with the anticoagulant will be temporarily stored in a portable cooler containing frozen gel-packs and refrigerated (4°C) until same day shipment to the central coordinating centre's laboratory in Vancouver occurs. From these samples, the participants' serum C-reactive protein (CRP) levels will be determined using a commercially available high-sensitivity enzyme-linked immunosorbent assay (ELISA) kits (Alpha Diagnostics, San Antonion, Tx). Serum concentrations of IL-6, TNF-α, and other inflammatory mediators will also be measured using commercially available ELISA kits.

#### Spirometry

Spirometry will be performed for each participant at each visit in accordance with the guidelines from the American Thoracic Society [25]. A test session will consist of several repeated FVC maneuvers. Each maneuver will require the subject to take the deepest possible breath and exhale into a spirometer as hard, fast, and completely as possible. The spirometer will record the volume of air exhaled as a function of time. Each subject will perform at least three FVC maneuvers. For a maneuver to be acceptable, it needs to be a maximal exhalation free from cough, excessive hesitation, leak, obstructed mouthpiece, variable effort, or early termination. At the first visit, pre and post-bronchodilator measurements will be done. For follow-up visits, only pre-bronchodilator values will be measured.

#### Health status measurements

During each visit, a disease-specific health measure, St. George's Respiratory Questionnaire (SGRQ), will be administered by the study co-ordinator at each site [26]. The scores for each section including symptom score (frequency and severity), activity score (activities that cause or are limited by breathlessness), and impact score (social functioning, psychological disturbances resulting from airways disease), as well as a total score will be calculated using established methods [26].

### Outcomes

#### Primary endpoint

The primary endpoint of the study is serum CRP level. We anticipate that inhaled fluticasone alone or in combination with salmeterol will reduce CRP levels by at least 30% below that achieved by placebo. We will also determine whether combination therapy reduces CRP levels beyond that achieved by fluticasone mono-therapy.

#### Secondary endpoints

The secondary endpoints will include: 1) changes in other inflammatory mediators such as IL-6, TNF-α, and monocyte chemotactic protein (MCP-1); 2) changes in SGRQ scores; 3) changes in FEV_1_; and 4) rates of exacerbations

### Statistics

#### Main analysis

For the primary analysis, we will compare the changes in CRP at 4 weeks from baseline between those assigned to inhaled fluticasone alone or in combination with salmeterol and those assigned to placebo based on an intention-to-treat principle. We will use between group t-tests to determine whether the changes in CRP between the groups are statistically significant and a mixed effects model to adjust for any significant differences in the baseline characteristics of the study participants across the groups.

#### Secondary analyses

We will perform similar analysis on TNF-α, IL-6 and MCP-1 (as well as other important inflammatory molecules). We will use Pearson's correlation techniques (as well as adjusted linear regression) to determine whether changes in CRP levels correlate with changes in SGRQ scores. Previous studies indicate that SGRQ improves with fluticasone with or without salmeterol, independent of their effects on FEV_1 _[10]. We hypothesize that SGRQ may correlate with CRP.

#### Sample size calculation

We will enroll 250 participants with COPD for this study. This will permit 100 participants to be assigned to fluticasone and combination treatment groups, respectively, and 50 participants will be exposed to a placebo during the active treatment phase. We have calculated our sample size based on the following assumptions: 1) we anticipate that fluticasone relative to placebo will lower baseline CRP levels by ~1.5 mg/L with standard deviation of 3.5 mg/L; 2) we will perform between group comparison of CRP (from baseline) using independent two sample t-test at alpha of 0.05 (two-tailed) and a power of ≥80%.

#### Ethical considerations

The protocol and procedures as described above have received institutional approval from the University of British Columbia/Providence Research Ethics Board, as well as from all local institutional ethics review board of each participating site. The funding for the study is from GlaxoSmithKline (Mississauga, ON). The funding source has no role in the design, conduct, analysis, interpretation, or reporting of the study and will not have access to the raw data. All data will be housed at the James Hogg iCAPTURE Center in Vancouver, BC.

## Discussion

In this paper, we have reported the background, the rationale, and the study protocol for a unique multi-center randomized controlled trial whose main outcome is a biochemical measurement (i.e. CRP). The study will test the hypothesis that inhaled corticosteroids alone or in combination with a long-acting β_2_-adrenoceptor agonist will reduce CRP levels in stable COPD patients. To our knowledge, this is the first clinical trial in COPD whose main outcome is serum CRP. Additionally, this study will be able to determine the effects of these medications on other relevant inflammatory biomarkers and likely provide a solid biological rationale for the clinical effects of inhaled corticosteroids and combination therapy in COPD.

There are certain limitations of this trial. Firstly, the study will not measure any parameters of inflammation in the lung. We decided against collecting biological samples from the airways because: 1) a recently conducted and published randomized controlled trial demonstrated that combination therapy reduces airway inflammation [20,21]; 2) there remains considerable amount of controversy regarding the utility of exhaled condensates and induced sputum in assessing airway inflammation in COPD [28]; 3) more invasive measurements (e.g. bronchoscopy) would have significantly reduced the projected recruitment rate of participants, reducing the power of the study to detect relevant differences in CRP; and 4) the primary focus of this study was to assess the effects of combination and steroid therapy on systemic inflammation. Secondly, the study has a relatively short follow-up period. We decided against a longer period of follow-up for pragmatic reasons. In our previous pilot study, we observed significant changes in CRP with inhaled corticosteroids after only 2 weeks of treatment [15]. Additionally, we were concerned that follow-up longer than four weeks would be associated with increased drop-out rates, and exacerbations, reduced compliance with the study medications, and changes in medications (both pulmonary and nonpulmonary drugs) that may alter CRP levels. Thirdly, we cannot be certain that changes in CRP levels will necessarily result in significant changes in health outcomes of COPD patients, though there is a body of indirect evidence suggesting that they do [6,14,17]. Additional work is needed in future studies to establish a causal link between CRP and health outcomes in COPD.

In summary, the present study will determine whether inhaled fluticasone alone or in combination with salmeterol has any significant systemic anti-inflammatory effects. Since systemic inflammation has been linked with a variety of important complications in COPD including peripheral muscle dysfunction, cachexia, cardiovascular complications and cancer, if these medications do indeed reduce systemic inflammation, this will provide an important biological mechanism by which they improve health outcomes in COPD. Additionally, this study will serve as an important template for future studies, linking systemic inflammation with clinical outcomes in COPD.

## Abbreviations

COPD = chronic obstructive pulmonary disease

CRP = C-reactive protein

TNF = tumor necrosis factor

IFN = interferon

IL = interleukin

FEV_1 _= forced expiratory volume in one second

FVC = forced vital capacity

SGRQ = Saint George's Respiratory Questionnaire

MCP-1 = monocyte chemoattractant protein-1

ELISA = enzyme-linked immunosorbent assay

ON = Ontario

## Competing interests

DDS & SFP have received honoraria for speaking engagements and research funding from GlaxoSmithKline (GSK).

## Authors' contributions

All of the authors contributed to the manuscript and to the project. DDS & SFP conceived the study, wrote the initial protocol, and obtained funding.

## Pre-publication history

The pre-publication history for this paper can be accessed here:
